# Radiomic signature based on CT imaging to distinguish invasive adenocarcinoma from minimally invasive adenocarcinoma in pure ground-glass nodules with pleural contact

**DOI:** 10.1186/s40644-020-00376-1

**Published:** 2021-01-06

**Authors:** Yining Jiang, Siyu Che, Shuangchun Ma, Xinyan Liu, Yan Guo, Ailian Liu, Guosheng Li, Zhiyong Li

**Affiliations:** 1 Department of Radiology, The First Affiliated Hospital of Dalian Medical University, Dalian, Liaoning Province People’s Republic of China; 2 GE Healthcare, Shenyang City, Liaoning Province China; 3 Department of Pathology, The First Affiliated Hospital of Dalian Medical University, Dalian, Liaoning Province People’s Republic of China

**Keywords:** Pure ground-glass nodules (pGGN), Pleural contact, Invasive adenocarcinoma (IAC), Minimally invasive adenocarcinoma (MIA), Radiomics, Computed tomography (CT)

## Abstract

**Background:**

Pure ground-glass nodules (pGGNs) with pleural contact (P-pGGNs) comprise not only invasive adenocarcinoma (IAC), but also minimally invasive adenocarcinoma (MIA). Radiomics recognizes complex patterns in imaging data by extracting high-throughput features of intra-tumor heterogeneity in a non-invasive manner. In this study, we sought to develop and validate a radiomics signature to identify IAC and MIA presented as P-pGGNs.

**Methods:**

In total, 100 patients with P-pGGNs (69 training samples and 31 testing samples) were retrospectively enrolled from December 2012 to May 2018. Imaging and clinical findings were also analyzed. In total, 106 radiomics features were extracted from the 3D region of interest (ROI) using computed tomography (CT) imaging. Univariate analyses were used to identify independent risk factors for IAC. The least absolute shrinkage and selection operator (LASSO) method with 10-fold cross-validation was used to generate predictive features to build a radiomics signature. Receiver-operator characteristic (ROC) curves and calibration curves were used to evaluate the predictive accuracy of the radiomics signature. Decision curve analyses (DCA) were also conducted to evaluate whether the radiomics signature was sufficiently robust for clinical practice.

**Results:**

Univariate analysis showed significant differences between MIA (*N* = 47) and IAC (*N* = 53) groups in terms of patient age, lobulation signs, spiculate margins, tumor size, CT values and relative CT values (all *P* < 0.05). ROC curve analysis showed, when MIA was identified from IAC, that the critical value of tumor length diameter (TLD) was1.39 cm and the area under the ROC curve (AUC) was 0.724 (sensitivity = 0.792, specificity = 0.553). The critical CT value on the largest axial plane (CT-LAP) was − 597.45 HU, and the AUC was 0.666 (sensitivity = 0.698, specificity= 0.638). The radiomics signature consisted of seven features and exhibited a good discriminative performance between IAC and MIA, with an AUC of 0.892 (sensitivity = 0.811, specificity 0.719), and 0.862 (sensitivity = 0.625, specificity = 0.800) in training and testing samples, respectively.

**Conclusions:**

Our radiomics signature exhibited good discriminative performance in differentiating IAC from MIA in P-pGGNs, and may offer a crucial reference point for follow-up and selective surgical management.

**Supplementary Information:**

The online version contains supplementary material available at 10.1186/s40644-020-00376-1.

## Background

The practicability of a new classification system for lung adenocarcinoma (ADC) was proposed by the International Association for Study of Lung Cancer, American Thoracic Society and the European Respiratory Society (IASLC, ATS, ERS) in 2011 [1]. Later in 2015, it was revised and adopted by the World Health Organization (WHO) [2]. ADC classification includes adenocarcinoma in situ (AIS), minimally invasive adenocarcinoma (MIA) and invasive adenocarcinoma (IAC). Patients with MIA survive well, postoperative recurrence and lymph node metastasis are rare, and the 5 year survival rate is close to 100%, whereas in contrast, an IAC prognosis is not as good [3–6]. Furthermore, studies have shown that pure ground-glass nodule (pGGNs), pathologically suspected to be AIS or MIA, require close follow-up or limited resection (segmental or wedge resection), while lobectomy is considered the standard surgical treatment for IAC [7].

Lung pGGN is a non-specific sign on thin-slice computed tomography (TSCT). The WHO recommended that if a biopsy indicated a pattern of lepidic growth with a pGGN on CT, AIS or MIA was proposed [2]. Previous studies have suggested that the proportion of IAC in pGGNs can be as high as 30.42–48.82% [8–11]. However, studies have also suggested that owing to limited CT image resolution (0.2–0.3 mm), a stromal or myofibroblastic invasion of an MIA ≤ 5 mm, or an IAC > 5 mm may be pGGN on high resolution CT [12].

Previous radiological studies suggested that the incidence of pleural contact signs in IAC groups were higher than MIA groups in ADC groups presented as pGGNs [13, 14]. In addition, studies using contrast analysis between High resolution CT (HRCT) signs and pathology concluded that pleural contact signs were significantly correlated with invasiveness, and more likely reminiscent of IAC [15, 16]. But pure ground - glass nodules with pleural contact (P-pGGNs) comprise not only the IAC, also part of the MIA [13, 14, 17], the proportion of which is as high as 42.86% [18]. Therefore, how can P-pGGNs be accurately assessed?

CT imaging plays an essential role in all phases of cancer management, including predictive evaluation. Zhao et al [19]*.* assessed 115 P-pGGNs cases and proposed that tumor shape, relative density and tumor-pleural relationships independently identified IAC from AIS/MIA. Receiver-operator characteristic (ROC) curve analysis for relative density confirmed sensitivity and specificity as 72.3 and 64.7%, respectively. Zhao et al. resulted in an unsatisfied diagnostic performance evaluation for these predictors of IA in P-pGGNs. By visually and subjectively assessing CT images to define the nodule’s type, observers may allow subtle but valid information to slip. In contrast to such qualitative inference, radiology fields that rely on imaging data have begun to benefit from radiomics. This approach recognizes complex patterns in imaging data and uses automatic or semi-automatic software to extract high-throughput radiographic images to quantify phenotypic intra-tumor heterogeneity in a non-invasive manner [20]. These features include descriptors of intensity distribution, spatial relationships between various intensity levels, texture heterogeneity patterns, descriptors of shape, and tumor relationships with the surrounding tissues (i.e., attachment to the pleural wall of the lung, or differentiation) [21]. Radiomics, as a tool that recognizes and evaluates histological subtypes, is currently under intensive focus. A recent study on pGGNs revealed that CT texture analysis, which assesses histograms, volumetrics, morphological and second-order texture features showed higher entropy and lower homogeneity and had the potential to distinguish AIS/MIA from IAC. The AUC of the ROC entropy curve, homogeneity and nodule size were 0.765, 0.671 and 0.712, respectively. By including mass, entropy and homogeneity into the logistic regression model, this improved the diagnostic accuracy (AUC = 0.962) when compared to conventional parameters alone, such as nodule size [22].

The nomogram is accepted as a reliable calculative instrument that visualizes risks posed by ADC [23]. Up to now, it is still unclear whether radiomic nomograms can be used to identify IAC and MIA in P-pGGNs. Thus, in this retrospective study, we developed and validated a radiomics signature for the identification of IAC and MIA as P-pGGNs in preoperative TSCT, which was later pathologically confirmed by surgery.

## Materials and methods

### Patients

This retrospective study was performed with institutional review board ethical approval. From December 2012 to May 2018, we identified 275 consecutive lung-pGGN patients (MIA; *N* = 167, IAC; *N* =108) who underwent preoperative chest TSCT, and were pathologically confirmed as single MIA and IAC after thoracic surgery. After screening, 100 cases with P-pGGN were finally included. The clinical characteristics of all P-pGGN cases were recorded (e.g., age, sex, smoking history, etc.).

Inclusion criteria: (A) patients having completed a lung TSCT scan 2 weeks before surgery; (B) pGGNs on lung window images (window width; 1500 HU, window level; − 600 HU), (C) single MIA and IAC pathologically confirmed by thoracic surgery, with accompanying histopathological specimens, (D) P-pGGNs located in the sub-pleural area displayed on preoperative TSCT, and defined as a pGGN attached or connected to the pleural surface, including visceral and interlobar pleura. Exclusion criteria: (A) patients having undergone tumor therapy (radiotherapy, chemotherapy, etc.), puncture biopsy, or surgical resection before the TSCT scan, (B) unavailable image archiving and communication systems, and (C) visible soft-tissue attenuation within the lesion, viewed on mediastinal window images (window width = 400 HU, window level = 40 HU).

### CT scan acquisition

All patients underwent chest CT examinations without intravenous contrast-media injection, with > 16 rows of spiral CT (GE Healthcare; GEHD 750; Somatom Perspective). Chest scans were performed on patients whose hands were at either side of the head in a supine position from the upper supraclavicular area to the lower adrenal area at deep inhalation and breath-holding moment. Scanning was performed in conventional helical mode, at a tube voltage of 120kVp, tube current 170–200 mA, slice thickness 5.0 mm, slice interval 5.0 mm, matrix 512× 512, bone algorithm for reconstruction, slice thickness 1.0–1.5 mm, and slice interval 1.0–1.5 mm.

### Pathological analysis

All pathological evaluations were performed by examining hematoxylin/eosin (HE) stained slides. These were prepared using formalin-fixed paraffin-embedded tissues with 0.4 cm thick sections, including the largest section of the tumor. All tumors were histologically evaluated by two experienced pathologists blinded to patient pathological information. The pathological type of each lesion was recorded. Pathological IAC and MIA diagnoses were performed according to the new ADC classification proposed by the IASLC/ATS/ERS in 2011. When opinions were divergent regarding morphology, discrepancies were resolved by consensus.

### Conventional image analysis

All raw CT images were retrieved on a picture archiving and communication system (PACS, DJ Health Union Systems Corporation), and observed by two experienced chest radiologists blinded to histological findings in the lung window (window width = 1500 HU, window level = − 600 HU). TSCT P-pGGN images were evaluated and the following imaging features were recorded.

#### Conventional morphological characteristics

(1) Tumor location: left superior lobe, left inferior lobe, right superior lobe, right middle lobe, right inferior lobe; (2) Shape: round and oval, irregular; (3) Tumor-lung interface: clear or not; (4) Lobulation signs: defined as a portion of the edge of the lesion; is wavy or fan-shaped; (5) Spiculate margin: defined as a thin line extending from the edge of the nodule to the lung parenchyma, but reaching the pleural surface; (6) Bubblelike appearance: defined as 1–3 mm cystic transparency of air attenuation within nodules; and (7) Air bronchogram: defined as lucency along the regular bronchial wall inside the P-pGGN.

#### Conventional quantitative CT features

(1) TLD on the largest axial plane (LAP): LAP was selected from the axial TSCT image on the lung window, and the maximum diameter on the LAP was determined as TLD; (2) Tumor short diameter (TSD) on the LAP. The TLD vertical diameter was determined on the LAP as the TSD; (3) Tumor vertical diameter (TVD) on the largest coronal plane (LCP) [24]: LCP was selected from the coronal TSCT image on the lung window, and the largest diameter on the LCP was measured as TVD; (4) CT value on the LAP (CT-LAP): the CT value on the LAP was measured as CT-LAP; (5) Relative CT value on the LAP (RCT-LAP) [19]: the normal lung density measured on the same plane with LAP (NLD-LAP) was divided by the CT-LA*P* value as RCT-LAP.

Measurement standards for conventional quantitative CT features: (1) The region of interest (ROI) of the P-pGGN was delineated by a regular curve. The ROI should include > 70% of the lesion area of the P-pGGN. (2) ROI measurements should avoid large vessels, bronchus and vacuoles, when there are bronchovascular bundles and vacuoles in the measurement layer. Sub-maximum layers should be selected when they cannot be completely avoided. (3) Selection of NLD-LAP: The same lobe and subpleural area at the same level of the lesion were selected. We completed ROI measurements by delineating the similar pulmonary microvascular attenuation and area as the CT-LAP measured.

### Radiomics analysis

#### Segmentation and radiomic feature extraction

All TSCT image layers were manually segmented, and radiomics feature were radiographically extracted using a free open-source software called 3D slicer (version 4.8.1) (https://www.slicer.org/). A total of 106 radiomics features were extracted automatically, and included shape (*N* = 13), Gray Level Dependence Matrix (GLDM; *N* = 14), Gray Level Co-occurrence Matrix (GLCM; *N* = 24), first order (*N* = 18), Gray Level Run Length Matrix (GLRLM; *N* = 16), Gray Level Size Zone Matrix (GLSZM; N = 16), and Neighboring Gray Tone Difference Matrix (NGTDM; *N* = 5), comprising seven categories.

#### Intra- and inter-observer agreements

Intra- and inter-observer agreements for feature extraction were evaluated using the intra- and inter-class correlation coefficient (ICC). Initially, 50 P-pGGN TSCT images were randomly selected, and ROI segmentation and feature extraction were independently completed by two blinded experienced radiologists. Observer one performed feature extraction on the CT image after an interval of no less than 30 days once more. Inter-observer agreement was assessed by comparing feature extraction measured by observer two, with feature extraction of observer one. The remaining image segmentation was measured by observer one, both independently and manually.

#### Radiomics feature selection and radiomics signature development

The dataset was assigned to either the training samples or testing samples in a 7:3 ratio. All cases in the training samples were used to select features and train the predictive model, while cases in the test cohort were used to independently evaluate the model’s performance. Before analyses, features were standardized by standardization. ICCs were calculated to determine inter- and intra-observer agreement, and features with ICCs > 0.75 were retained. The least absolute shrinkage and selection operator (LASSO) method was used for regression assessment of high-dimensional data. LASSO was used to derive the most useful predictive features. Optimal features were selected according to the AUC. In LASSO, a 10-fold cross-validation was performed to choose the optimal hyperparameter log (λ), with mean square error as a criterion (the smaller the better). The radiomics signature (Rad-score) was calculated based on the sum of selected features weighted by their corresponding coefficients to predict IAC before surgery for each patient. A 10-fold cross-validation in the training samples was also performed to evaluate the performance and reliability of our model. A logistics model was built from optimal feature subsets of the training sample.

#### Evaluation of the radiomics signature

**Fig. 1:**
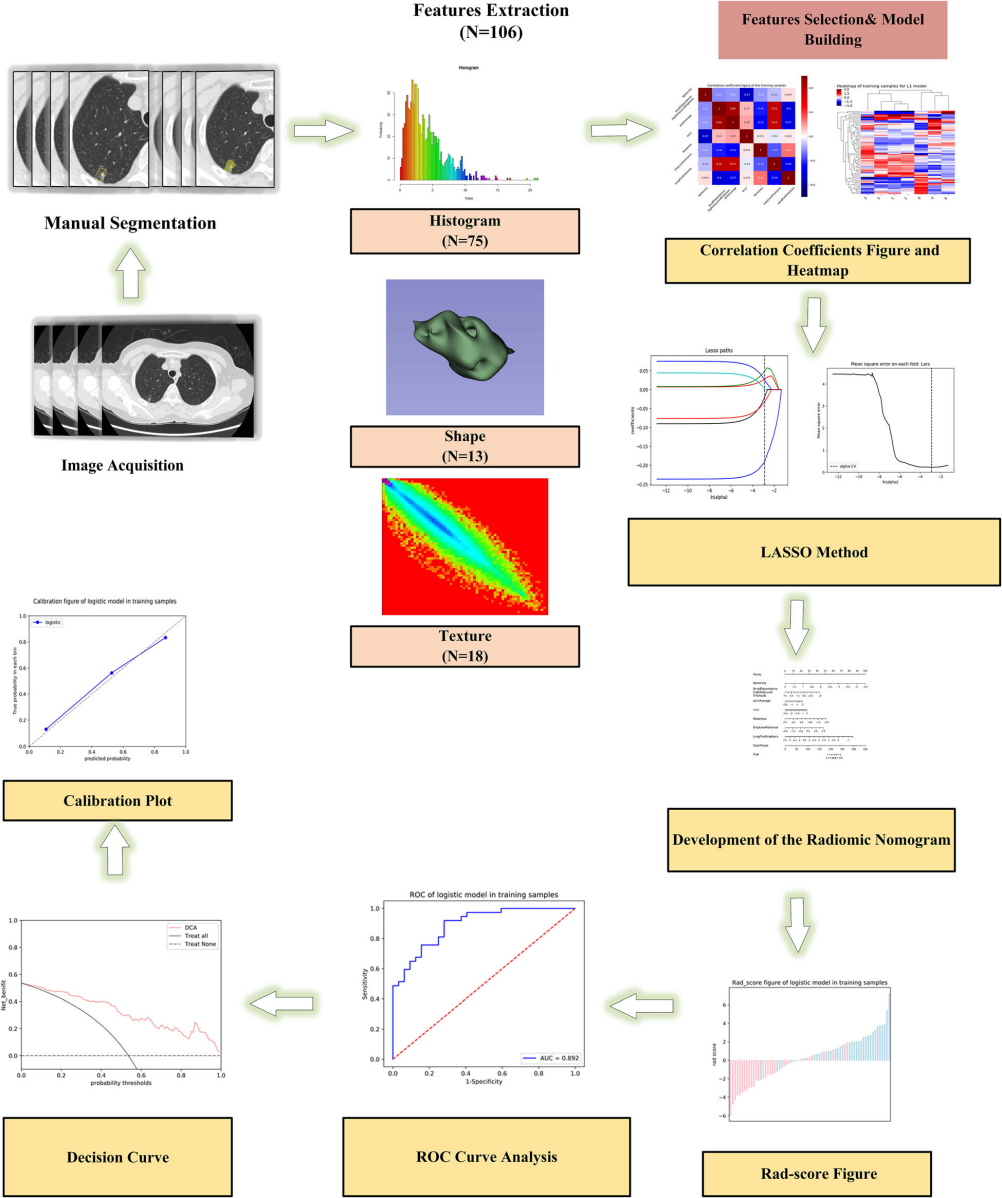
Flow diagram showing original imaging to radiomics-model building. LASSO; the least absolute shrinkage and selection operator method; AUC, the area under the ROC curve; ROC, receiver operating characteristics

The AUC of the ROC curve was used to evaluate the predictive accuracy of the radiomics model, in both training and testing samples. A calibration curve was used to demonstrate the calibration degree, which reflected consistency between predicted and observed IAC risks. Decision curve analysis (DCA) was conducted to evaluate the clinical usefulness of the radiomics model (Fig. 1).

### Statistical analysis

The Kolmogorov-Smirnov test evaluated whether variables were normally distributed. Variables were described as the mean ± standard deviation (SD) for normal distributions, and described as the median and quartile for non-normal distributions. T-tests were used for normally distributed variables, and the Mann–Whitney U test was used for non-normally distributed variables. A Pearson χ2 test or Fisher exact test was used to test differences between groups in terms of tumor location, shape, tumor-lung interface, lobulation signs, spiculate margins, bubblelike appearance and air bronchograms. ROC curves were plotted to assess the performance of conventional quantitative CT features in differentiating IAC from MIA groups. Accuracy, sensitivity, specificity and AUC were also calculated. LASSO methods were performed using the “glmnet” package. A calibration curve was performed to evaluate the predictive accuracy of the radiomics signature. DCA was conducted to evaluate whether the radiomics signature was sufficiently robust for clinical practice. All statistical analyses were performed using SPSS (version 26.0, IBM, Armonk, NY, USA), R 3.5.1 and Python 3.5.6. A two-tailed *P*-value < 0.05 indicated statistical significance.

## Results

### Baseline characteristics

One hundred eligible single P-pGGNs, of which 47 were MIA (47%) and 53 were IAC (53%) were included. None of the lesions was associated with any mediastinal lymph node metastases. Twenty-nine (29%) males and 71 (71%) females participated, with a median age of 60.50 years (50.00–66.00 years). The IAC group was older than the MIA group (63.00, 56.00–67.00 vs. 55.50, 44.50–63.50, *P* = 0.006). Most of the underlying diseases were localized and mild, and there were more underlying diseases in the IAC group than in the MIA group (IAC identified:bronchitis,1;pulmonary bullae 7;emphysema 7;MIA identified: tuberculosis 1). We observed no statistical differences in terms of smoking history, gender and statistical differences in terms of underlying disease between groups (Table 1).

**Table 1:** Patient P-pGGN characteristics

Variable	IAC(*N*=53)	MIA(*N*=47)	Total	*P* value
Age Median (25th to 75th percentile) years	63.00 (56.00~67.00)	55.50 (44.50~63.50)	60.50 (50.00~66.00)	0.006
Sex/Male No. (%)	16 (30.19)	13 (27.66)	29 (29.00)	0.781
Smoking history, No. (%)				0.719
Ever smokers	5 (9.43)	3 (6.38)	8 (8.00)	
Never smokers	48 (90.57)	44 (93.62)	92 (92.00)	
Underlying disease				0.000
Positive				
Bronchitis	1 (1.00)	0 (0.00)	1 (1.00)	
Pulmonary bullae	7 (7.00)	0 (0.00)	7 (7.00)	
Emphysema	7 (7.00)	0 (0.00)	7 (7.00)	
Tuberculosis	0 (0.00)	1 (1.00)	1 (1.00)	
Negative	38 (38.00)	46 (46.00)	84 (84.00)	

*IAC* Invasive adenocarcinoma, *MIA* Minimally invasive adenocarcinoma

### Conventional morphological characteristics

Lobulation signs and spiculate margin indications in the IAC group were significantly higher than the MIA group (54.72% vs. 25.53%, *P* = 0.003; 60.37% vs. 29.79%, *P* = 0.002). Other objective morphological characteristics (i.e., tumor location, shape, tumor-lung interface, bubblelike appearance, air bronchogram) showed no statistical differences between groups (*P* > 0.05) (Table 2).

**Table 2:** Morphological index analyses

Morphological index	IAC	MIA	Total	*P* value
Tumor location, No. (%)				0.671
Left lung				
Superior lobe	15 (28.30)	11 (23.41)	26 (26.00)	
Inferior lobe	6 (11.32)	9 (19.15)	15 (15.00)	
Right lung				
Superior lobe	17 (32.08)	16 (34.04)	33 (33.00)	
Middle lobe	3 (5.66)	4 (8.51)	7 (7.00)	
Inferior lobe	12 (22.64)	7 (14.89)	19 (19.00)	
Shape, No. (%)				0.536
Round and oval	17 (32.08)	18 (38.30)	35 (35.00)	
irregular	36 (67.92)	29 (61.70)	65 (65.00)	
Tumor-lung interface(clear), No. (%)	31 (58.49)	25 (53.19)	56 (56.00)	0.687
Lobulation sign, No. (%)				0.003
Positive	29 (54.72)	12 (25.53)	41 (41.00)	
Negative	24 (45.28)	35 (74.47)	59 (59.00)	
Spiculate margin, No. (%)				0.002
Positive	32 (60.37)	14 (29.79)	46 (46.00)	
Negative	21 (39.62)	33 (70.21)	54 (54.00)	
Bubblelike appearance, No. (%)				0.159
Positive	18 (33.96)	10 (21.28)	28 (28.00)	
Negative	35 (66.04)	37 (78.72)	72 (72.00)	
Air bronchogram, No. (%)				0.446
Positive	11 (20.75)	7 (14.89)	18 (18.00)	
Negative	42 (79.25)	40 (85.11)	82 (82.00)	

*IAC* Invasive adenocarcinoma, *MIA* Minimally invasive adenocarcinoma

### Conventional quantitative CT features

**Fig. 2:**
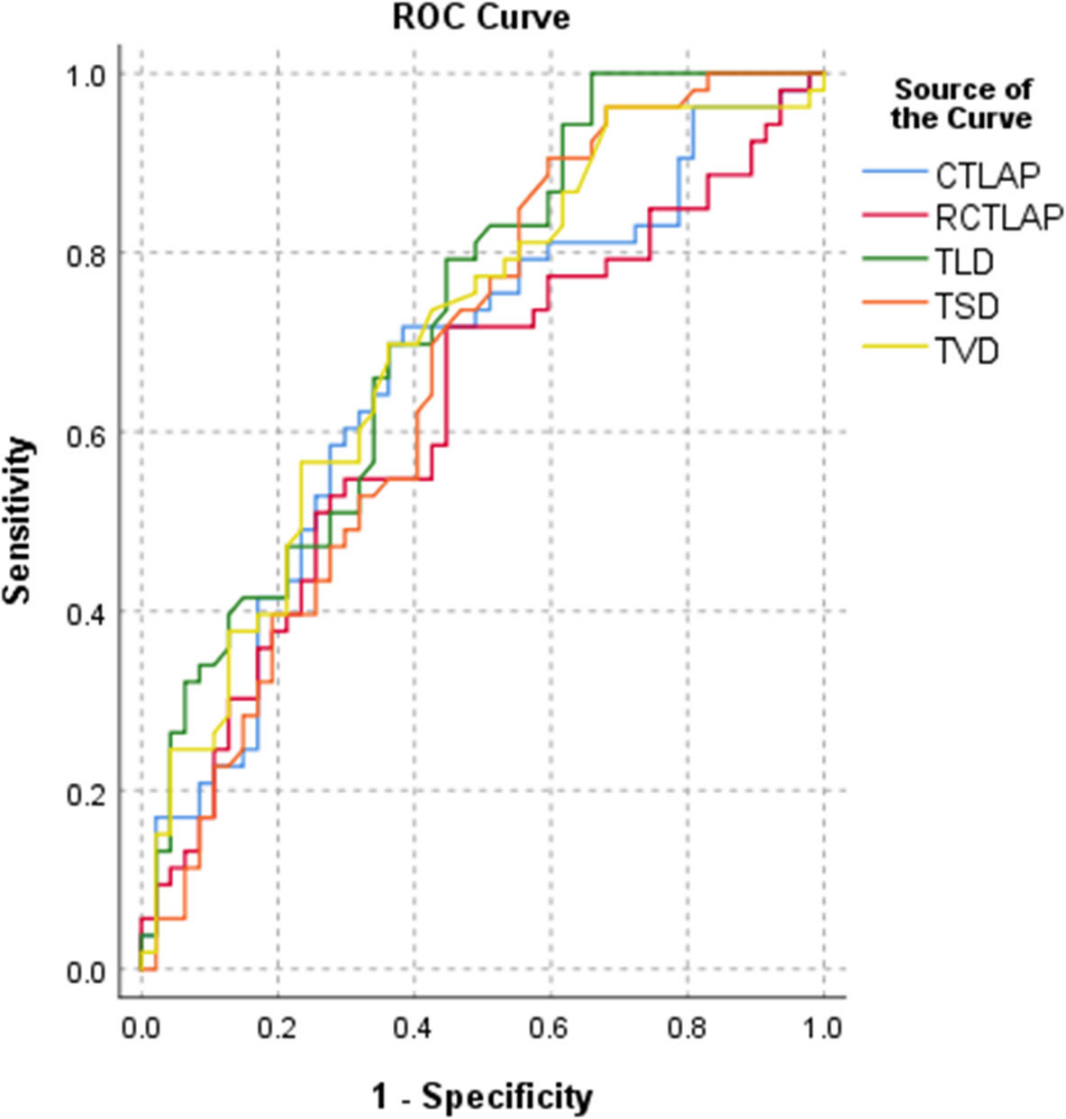
ROC curve analysis of TLD, TSD, TVD, CT-LAP and RCT-LAP, predicting IAC

**Fig. 3:**
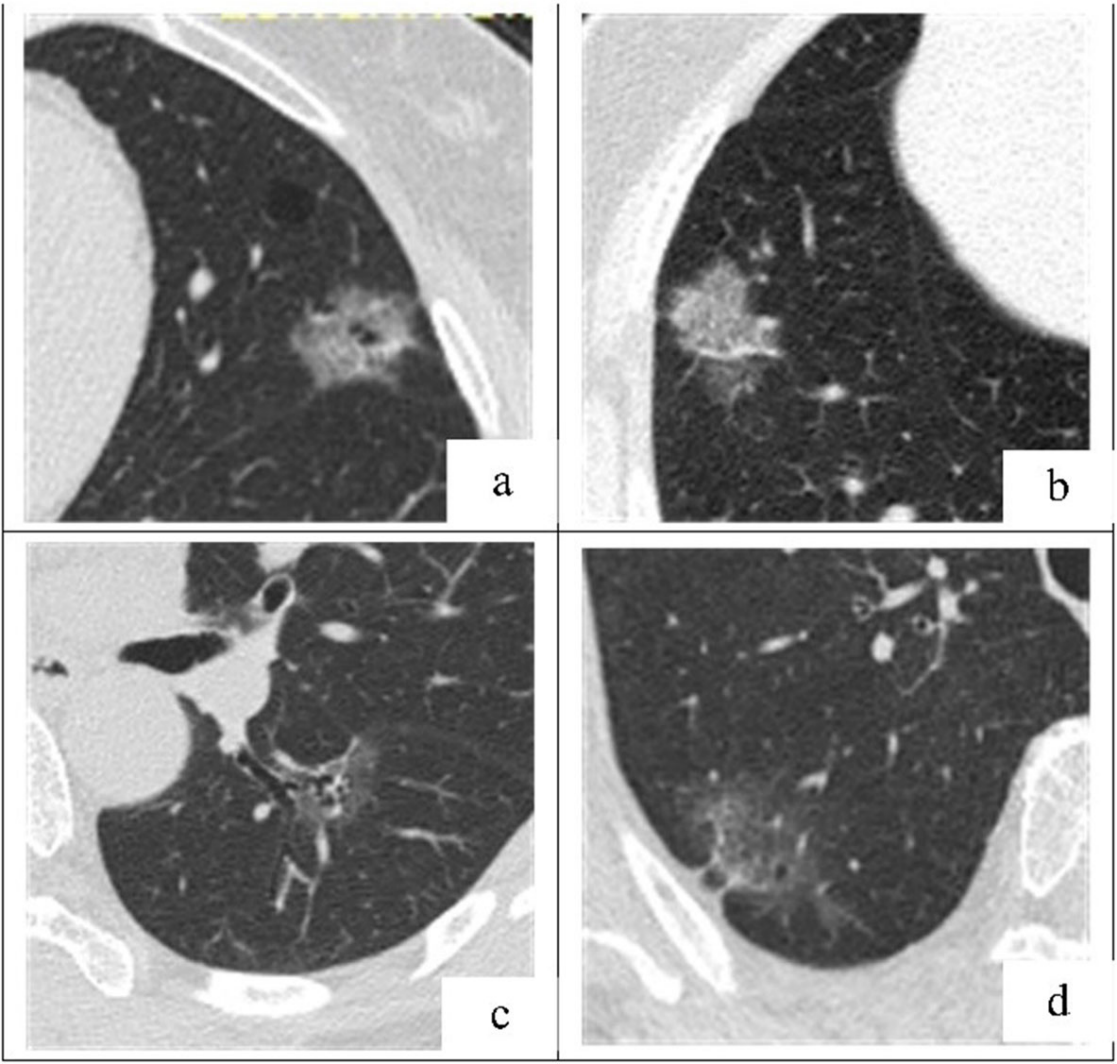
**a-d**. Representative axial TSCT images of P-pGGNs in four patients, connected to the visceral pleural surface. The longer TLD, higher CT-LAP, lobulation signs and bubblelike-appearance suggest P-pGGN invasiveness. **a** A 63-year-old female with a P-pGGN in the left superior lobe, diagnosed as IAC, showed a 2.32 cm-TLD, lobulated, bubblelike-appearance, and − 467.31 HU-CT-LAP; **b** A 59-year-old female with a P-pGGN in the right superior lobe, diagnosed as IAC, showed a 3.09 cm-TLD lobulated and − 360.13 HU-CT-LAP; **c** A 57-year-old female with a P-pGGN in the left inferior lobe, diagnosed as MIA, showed a 2.36 cm-TLD, and − 630.43 HU-CT-LAP; **d** An 80-year-old female with a P-pGGN in the right superior lobe, diagnosed as MIA, showed a 4.16 cm-TLD, and − 662.7 HU-CT-LAP

Significant differences in TLD, TSD, TVD, CT-LAP and RCT-LAP between groups are shown (*P* < 0.05) (Table 3). The CT-LAP and RCT-LAP of the IAC group were higher than the MIA group (− 560.94 HU vs. -620.45 HU, *P* = 0.004; 1.54 vs. 1.40, *P* = 0.034). The TLD, TSD and TVD of the IAC group were also higher than the MIA group. ROC curve analysis showed that when identifying MIA from IAC, the TLD critical value was 1.39 cm and the AUC was 0.724 (sensitivity = 0.792, specificity = 0.553). The CT-LAP critical value was − 597.45 HU, and the AUC was 0.666 (sensitivity = 0.698, specificity = 0.638). The AUC for TSD, TVD and RCT-LAP ranged between 0.623–0.702 (Table 4, Figs. 2 and 3a-d).

**Table 3:** Conventional quantitative CT features analyses

Quantitative indicators	IAC	MIA	*P* value
TLD (cm) Median (25th to 75th percentile) cm	1.81 (1.40~2.56)	1.28 (0.85~1.87)	0.000
TSD (cm) Median (25th to 75th percentile)	1.49 (1.09~2.10)	1.09 (0.79~1.61)	0.004
TVD (cm) Median (25th to 75th percentile)	1.51 (1.23~2.13)	1.16 (0.79~1.42)	0.000
CT-LAP (HU) Median (25th to 75th percentile)	−560.94(−625.01~− 477.06)	−620.45(− 655.94~− 551.01)	0.004
RCT-LAP (HU) Median (25th to 75th percentile)	1.54 (1.38~1.72)	1.40 (1.32~1.55)	0.034
Normal lung density (HU) Median (25th to 75th percentile)	− 868.79(− 890.52~ −841.36)	− 877.43(− 899.33~− 849.76)	0.218

*TLD* Tumor length diameter on the largest axial plane, *TSD* Tumor short diameter on the largest axial plane, *TVD* Tumor vertical diameter on the largest coronal plane, *CT-LAP* CT value on the largest axial plane, *RCT-LAP* relative CT value on the largest axial plane, *NLD-LAP* normal lung density measured on the same plane with LAP, *HU* Hounsfield units

**Table 4:** ROC curve analyses

Quantitative indicators	AUC	p	Critical value	Sensitivity	Specificity	Youden index
TLD (cm)	0.724	0.000	1.39	0.792	0.553	0.345
TSD (cm)	0.669	0.004	0.91	0.906	0.404	0.310
TVD (cm)	0.702	0.000	1.28	0.698	0.638	0.336
CT-LAP(HU)	0.666	0.004	−597.45	0.698	0.638	0.336
RCT-LAP	0.623	0.034	1.43	0.717	0.553	0.270

*TLD* Tumor length diameter on the largest axial plane, *TSD* Tumor short diameter on the largest axial plane, *TVD* Tumor vertical diameter on the largest coronal plane, *CT-LAP* CT value on the largest axial plane, *RCT-LAP* relative CT value on the largest axial plane, *NLD-LAP* normal lung density measured on the same plane with LAP, *HU* Hounsfield units

### Radiomics feature selection and signature development

**Fig. 4:**
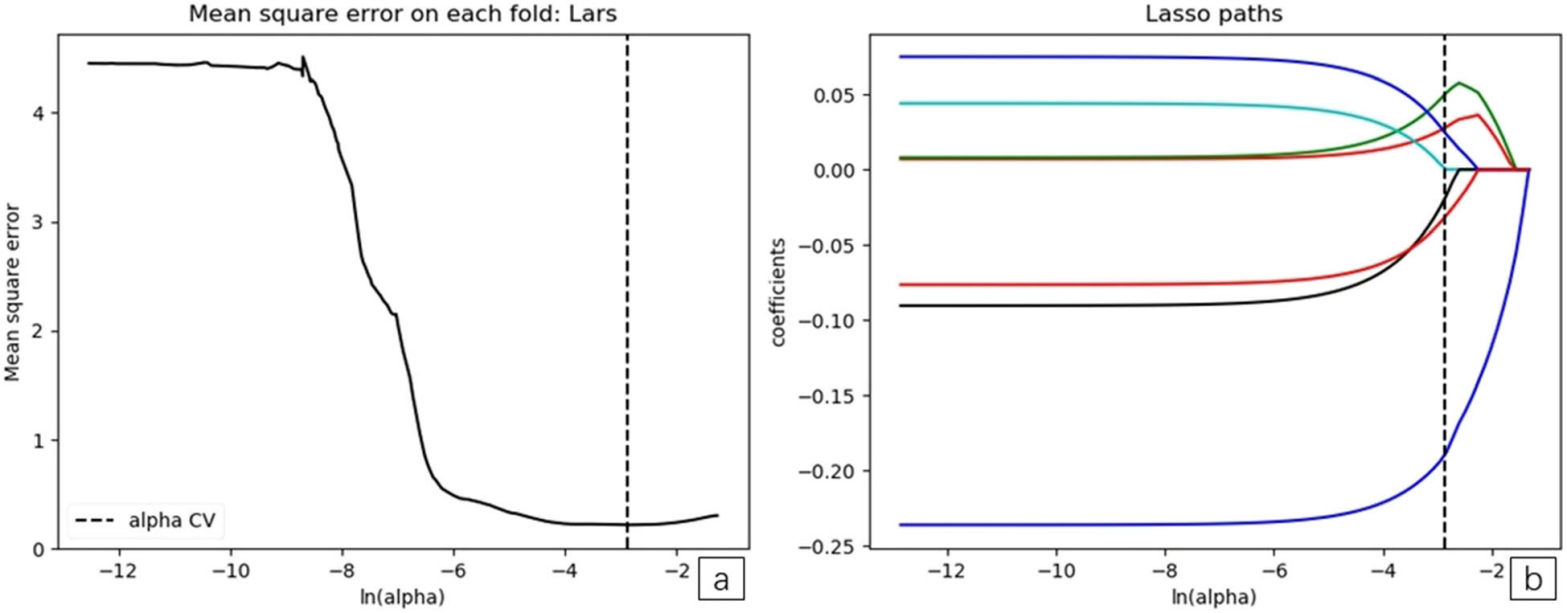
**a and b**. The least absolute shrinkage and selection operator (LASSO) binary logistic regression model for feature selection. The features retained were introduced into the LASSO regression model. First, a 10-fold cross-validation method was used to screen the LASSO regression model hyperparameter (λ) to select the model with the smallest error (λ). A vertical line was drawn at the selected value using 10-fold cross-validation, where optimal λ resulted in seven non-zero coefficients

We completed ROI feature extraction and consistency tests within and between observers. Intra-observer ICCs ranged from 0.475–0.995, and there were 13 features ICC < 0.750. Inter-observer ICCs ranged from 0.278–0.992, and inter-observer showed 17 features ICC < 0.750. When ICC > 0.750, the features were repeatable. Nineteen features of poor repeatability were eliminated. The remaining 87 radiomics features showed favorable intra- and inter-observer reproducibility and consistent feature extraction. The detailed results of intra- and inter-observer ICCs are listed in Additional file 1: Table 1. In LASSO regression analysis using 10-fold cross-validation, the best alpha was 0.036. At this value, 87 radiomics features were reduced to seven potential predictors with non-zero coefficients. Log (λ) changes from – 10 to 0, features that participated into the model is reduced, and the absolute values of the coefficients of the variables also shrink toward zero (Fig. 4a and b).

**Fig. 5:**
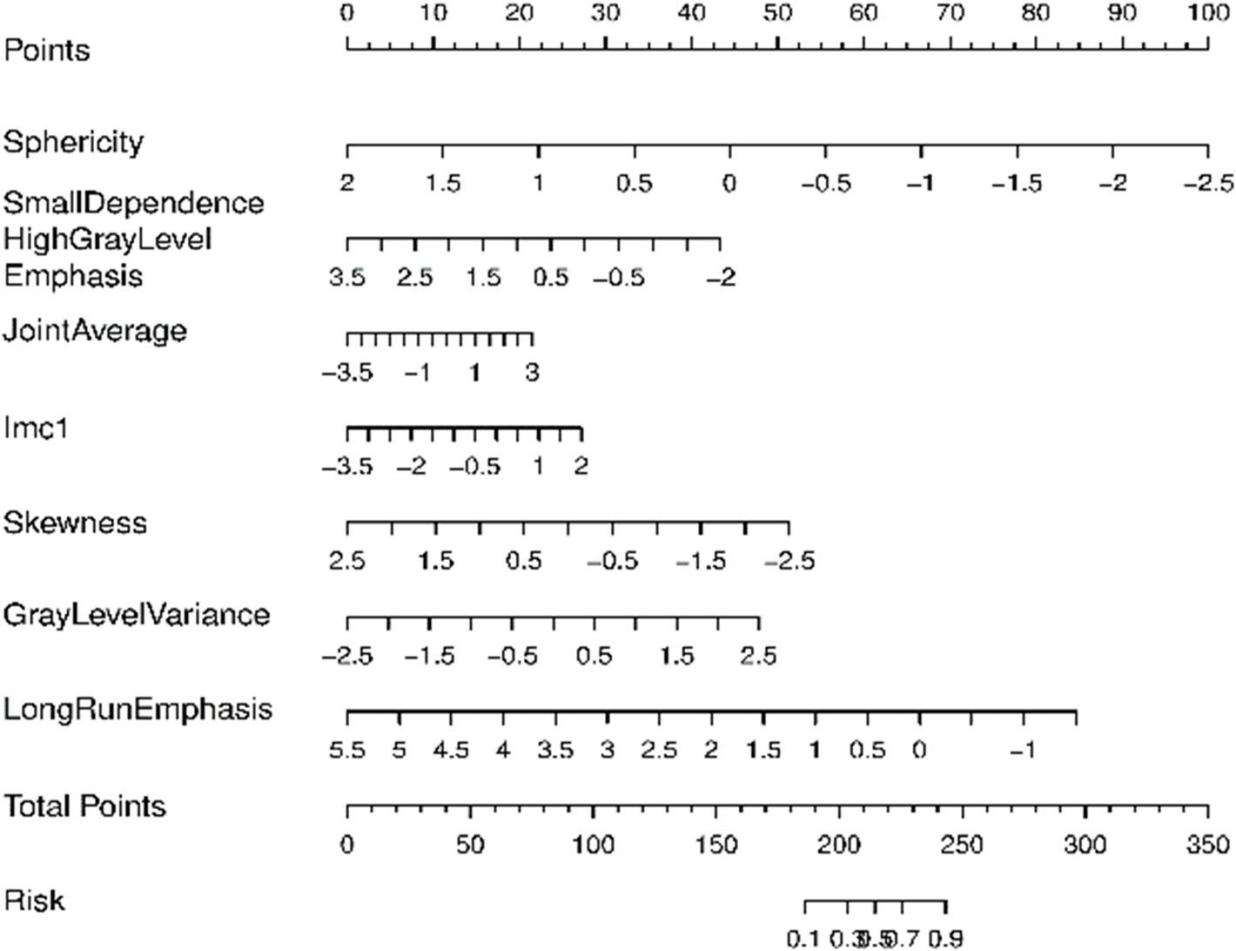
Nomogram plot of the logistic model in training samples. To assess the probability of IAC in P-pGGNs, patient values were marked at each feature axis, a straight line was drawn perpendicular to the point axis, and a corresponding point for each feature was obtained. All points were summed for all features. Next, the sum was marked on the total points axis, and a straight line drawn perpendicular to the risk axis

These features were enrolled into signatures, including shape features (*N* = 1): Sphericity; GLDM(N = 1): Small Dependence High Gray Level Emphasis; GLCM; (*N* = 2): Joint Average, Imc1; first order feature(N = 1): Skewness; GLRLM(N = 2): Gray Level Variance 1, Long Run Emphasis. Definitions of these radiomics features are available (Additional file 1). A logistic-model-based nomogram was also performed. According to these radiomic nomograms, each predictive feature was assigned a weighted number of points (Fig. 5). The total number of points for each patient was calculated using the nomogram, and was associated with an estimated IAC probability, present as P-pGGNs. The calculation formula was:

$$ \mathrm{Rad}\ \mathrm{score}=0.189-1.701\times \mathrm{Sphericity}-0.603\times \mathrm{Small}\ \mathrm{Dependence}\ \mathrm{High}\ \mathrm{Gray}\ \mathrm{Level}\ \mathrm{Emphasis}+0.253\times \mathrm{Joint}\ \mathrm{Average}+0.378\times \mathrm{Imc}1-0.786\times \mathrm{Skewness}+0.732\times \mathrm{Gray}\ \mathrm{Level}\ \mathrm{Variance}.1-0.925\times \mathrm{Long}\ \mathrm{Run}\ \mathrm{Emphasis}. $$

**Fig. 6:**
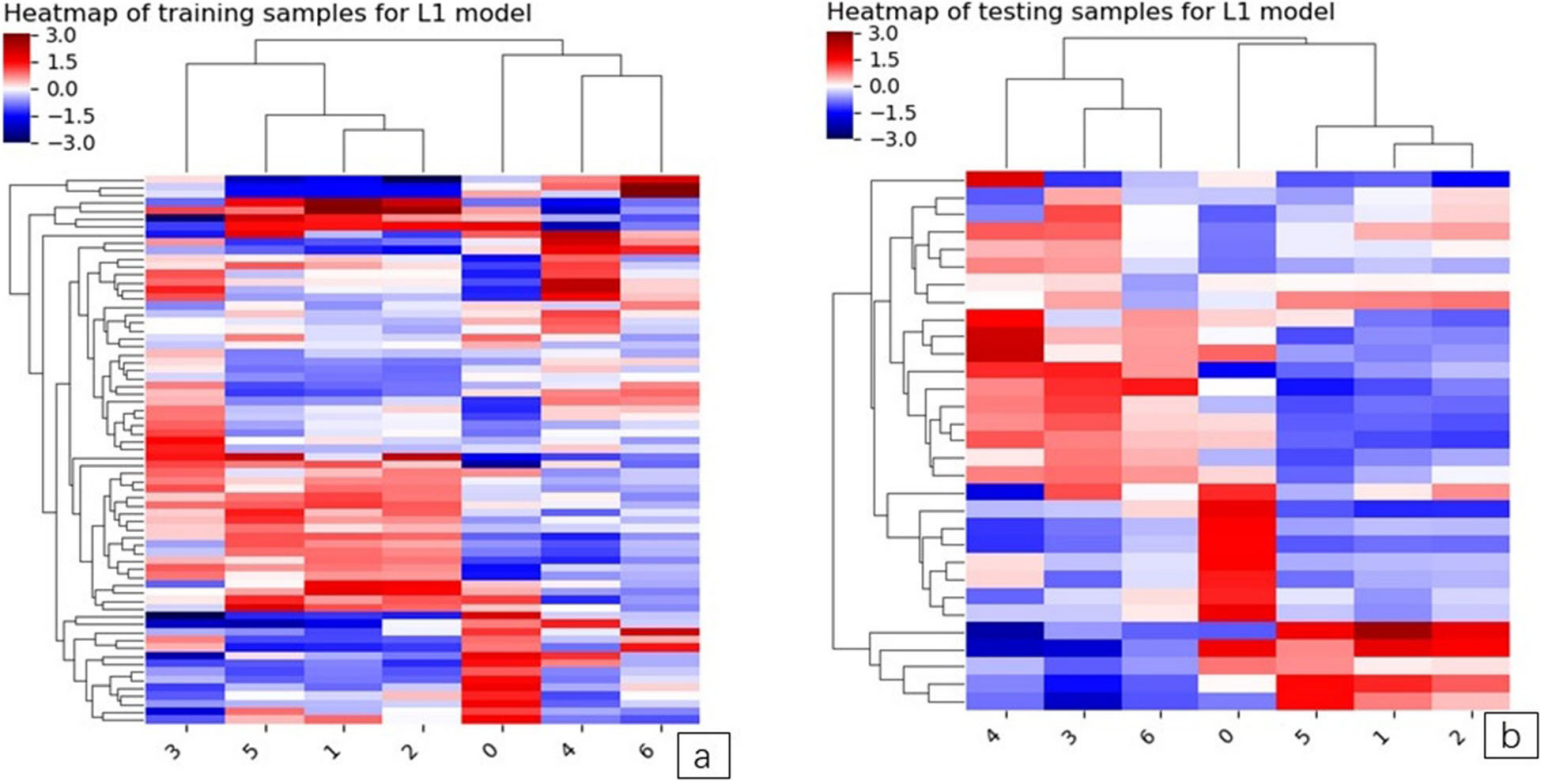
**a and b**. The left image is the model heatmap of training samples. The right shows the model heatmap in testing samples. Unsupervised clustering of patients (*N* = 61) on the y-axis and expression of radiomics features (*N* = 7) on the x-axis reveals clusters of patients with similar radiomics expression patterns. There was a significant association of radiomics feature expression patterns with the two different P-pGGN groups. The indicators corresponding to the dark red squares are more predictive

**Fig. 7:**
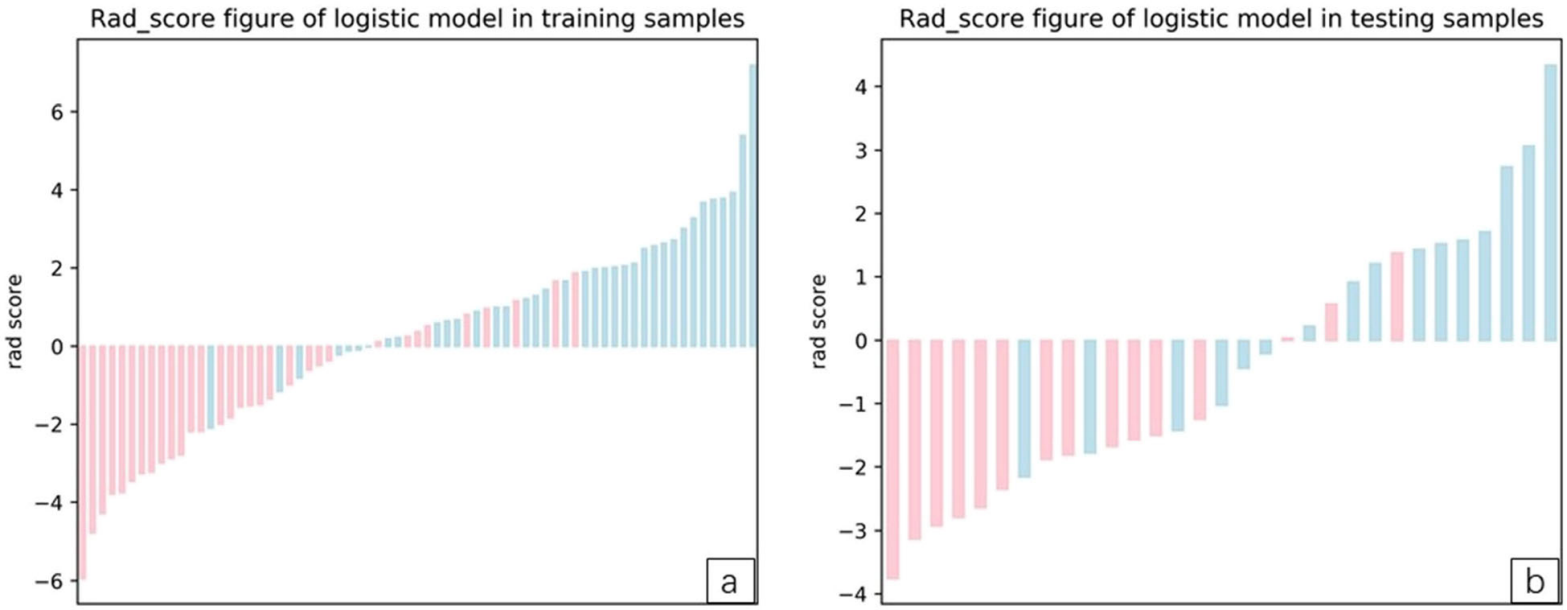
**a and b** The left image shows the Rad-score figure of the training samples, and the right shows the Rad-score figure of the testing samples. The red and blue bars represent the two samples. The scale 0 represents the cut-off value. If the scale 0 separates the red bar from the blue bar, the signature identification ability exhibits good performance

The heatmap and rad-score of the features in the model are described (Figs. 6 and 7a and b).

### Validation of the radiomics signature

**Fig. 8:**
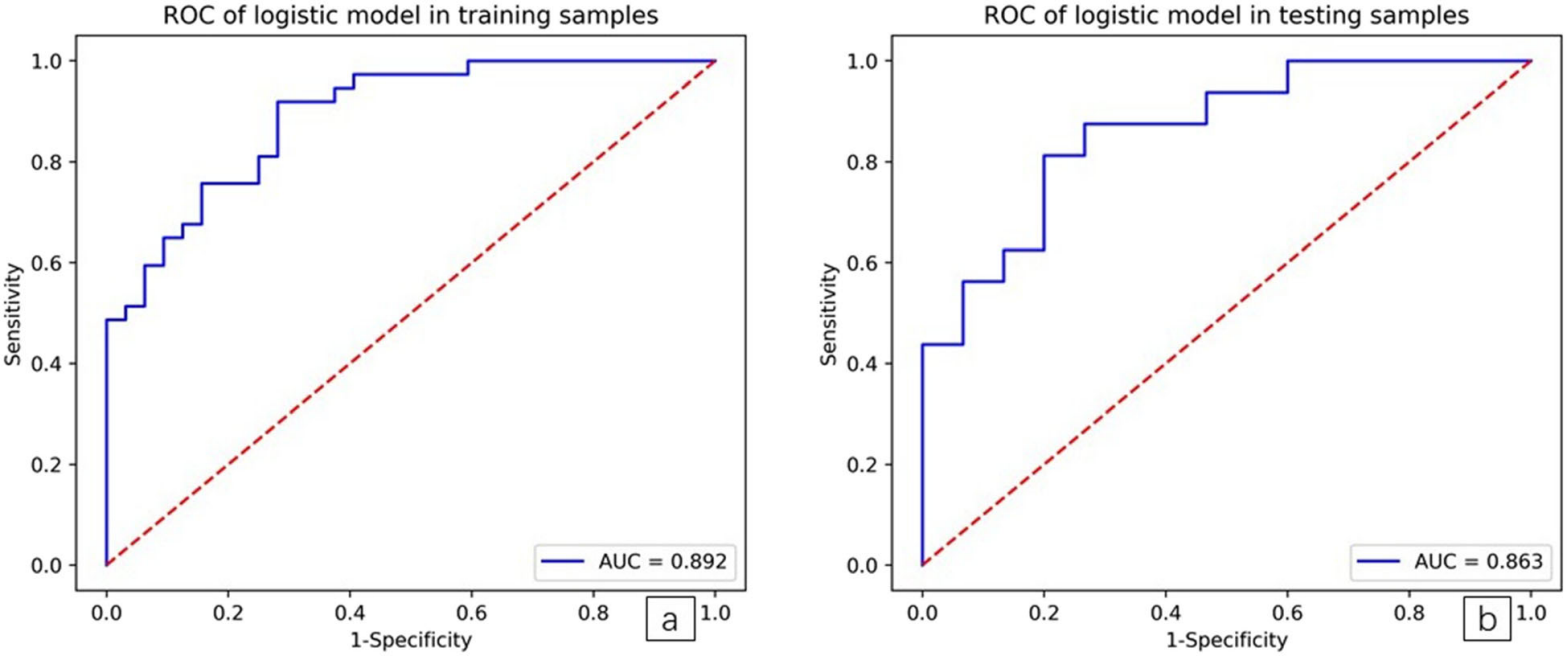
**a and b**. Receiver operating characteristic (ROC) curves for training and testing samples. The radiomic features predictive signature predicted the preoperative discrimination of IAC and MIA as P-pGGNs. (The AUC for training samples was 0.892 and the AUC for testing samples was 0.863)

**Fig. 9:**
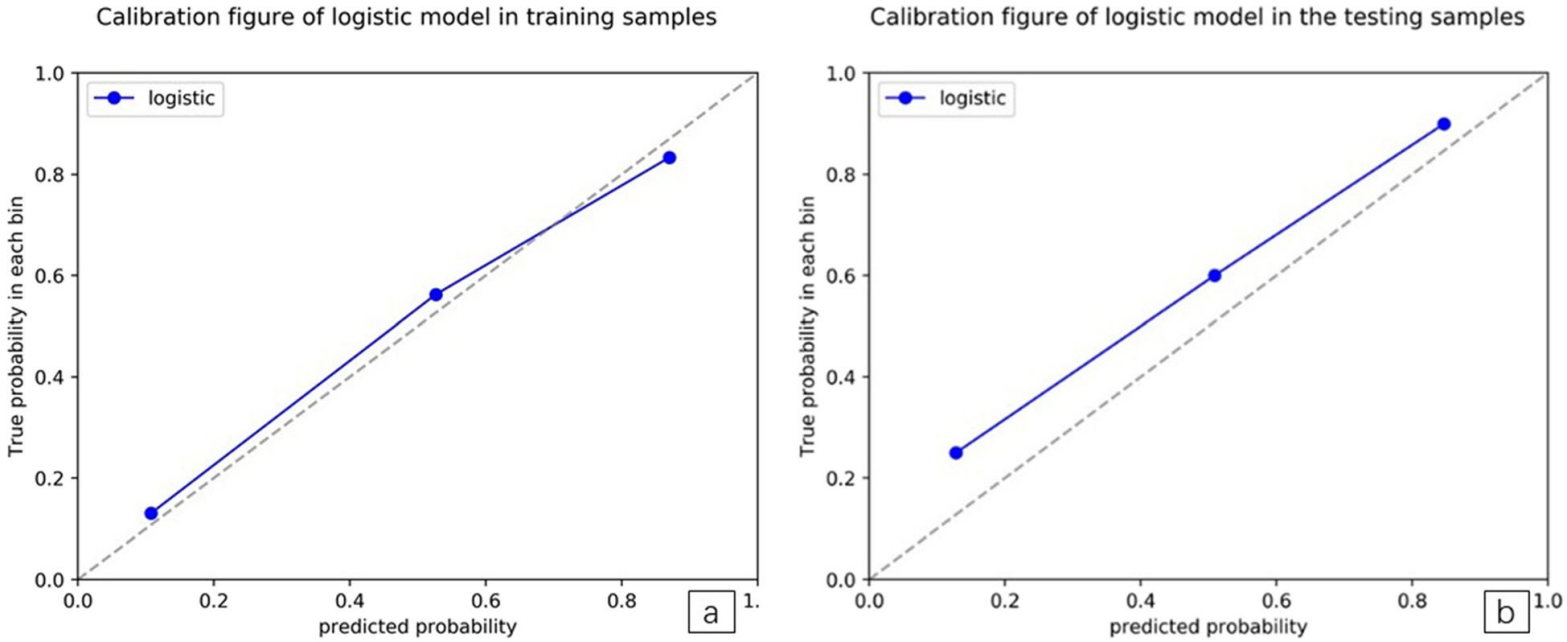
**a and b.** Calibration plots of radiomic models for training and testing samples. Calibration curves evaluated the correspondence between predicted and observed probabilities. The closer the solid line to the grey dotted line, the better the prediction model

ROC curves showed that the LASSO regression prediction model, which was based on a combination of seven radiomics features, had good performance and generality in distinguishing IAC and MIA as P-pGGNs. In terms of discriminating analysis, the signatures had AUCs of 0.892 (sensitivity = 0.811, specificity = 0.719) and 0.862 (sensitivity = 0.625, specificity = 0.800) in train and test samples, respectively (Table 5, Fig. 8a and b). The AUCs in test samples declined slightly when compared with train samples, but still had a satisfactory classification performance. Three IAC nodules were underestimated by the radiomics signature, and all six cases were predicted as MIA (Fig. 7a and b). Calibration curves demonstrated that IAC probabilities were consistent between predictions and observations in both train and test samples (Fig. 9a and b).

**Table 5:** Evaluation of the logistic model in training and testing samples

Item	Train	Test	Total
Patients, No.			
IAC	37	16	53
MIA	32	15	47
Accuracy	0.768	0.71	
Precision	0.769	0.769	
AUC	0.892	0.863	
Sensitivity	0.811	0.625	
Specificity	0.719	0.800	
positive prediction	0.769	0.769	
negative prediction	0.767	0.667	

*MIA* Minimally invasive adenocarcinoma, *IAC* Invasive adenocarcinoma, *AUC* area under the ROC curve

**Fig. 10:**
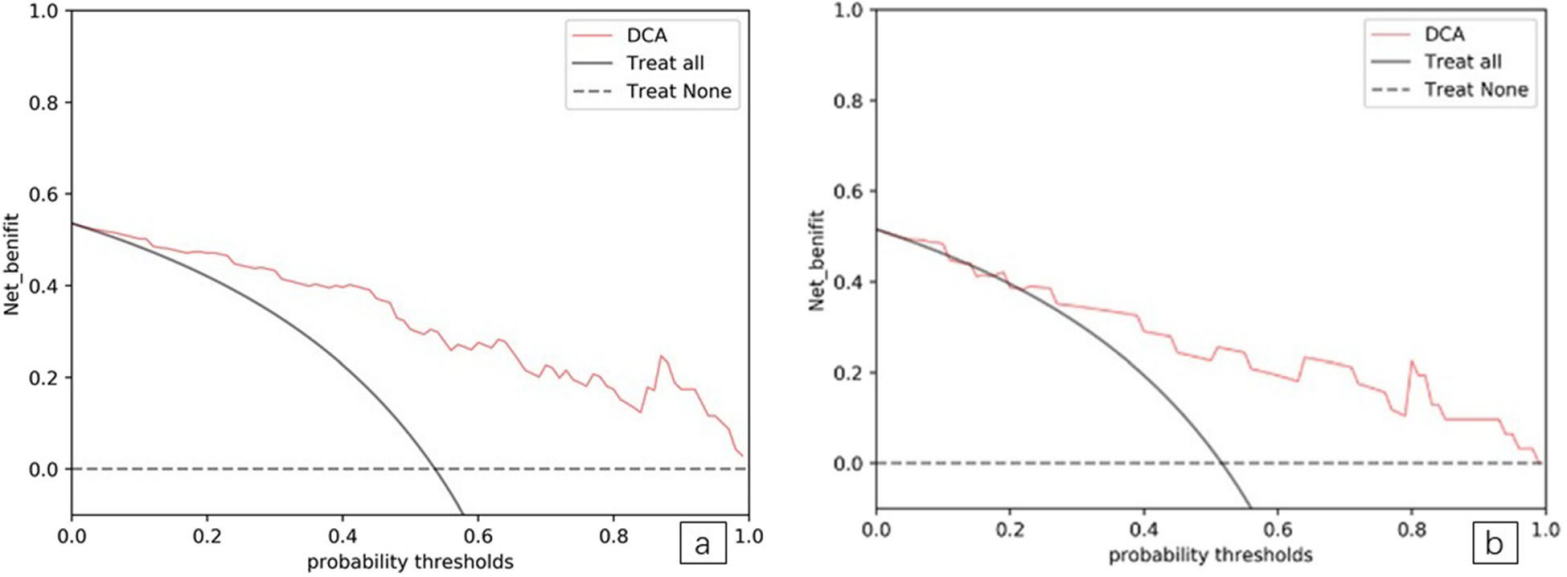
**a and b.** Decision curve of radiomic signature in training and testing samples. The net benefit is shown on the y-axis, and the probability threshold is shown on the x-axis. The radiomics model (red line) generated a net benefit in both training and testing samples

“True” positive and weighted false-positive rates were calculated across different threshold probabilities in the validation set, to determine the net benefit. Specifically, the weighting factor was defined as the specific value of the threshold probability, divided by 1, minus the threshold probability. A higher true-positive rate and a relatively low false-positive rate was suggested by a high net benefit. Finally, we used a DCA curve to assess whether this model would help with clinical treatment strategies. When the threshold probability varied from 0 to 1, according to the DCA, the radiomics model achieved a high net benefit, when compared with a “treat all” and a “treat none” strategy (Fig. 10a and b).

## Discussion

To the best our knowledge, the present research is the first to utilize a quantitative radiomics signature to differentiate IAC from MIA appearing as P-pGGNs. We systematically evaluated the conventionally morphological and quantitative CT features of enrolled P-pGGNs and processed 106 radiomics features, then a signature was developed, confirmed that CT-based imaging radiomics features could accurately predict the presence or absence of IAC from MIA in P-pGGNs. The AUC was 0.892 and 0.863 for the training and test samples, respectively.

Since the WHO adopted a new classification for ADC in 2015, researchers have continuously explored correlations between CT findings and tumor pathology. It has been accepted that pGGNs with pleural contact signs are predisposed to IAC [25, 26]. Our study revealed the occurrence probability of pleural contact signs in MIA was reached 28.14% (47/167) almost same as one report as 30.76% [27]. Therefore, the effective identification of MIA and IAC in P-pGGNs is clinically meaningful.

In our study, females and nonsmokers were associated with the P-pGGN group. Similarly, age was also associated with IAC. Patients with IAC tended to be older, while patients with MIA were younger. This was consistent with previous findings [28]. Morphological features from CT images are frequently used in routine clinical practice for the ADC with pGGNs differential diagnostic process [29]. Our study confirmed that a P-pGGN with lobulation and spiculate interface had the greater likelihood of being an IAC (54.72% vs. 25.53%, *P* = 0.003; 60.37% vs. 29.79%, *P* = 0.002),these results were similar to a previous study [15]. There were no differences in other signs, including irregular shape, bubblelike appearance and air bronchograms.

Conventionally, quantitative features from CT image can independently identify the pathological invasiveness of ADC. The nodule size is not ignorable indicators of risk for predicting P-pGGNs as IAC. According to ROC curve analysis, the AUC of the nodule size index, TLD was the highest, i.e., 0.724, and the critical value was 1.39 cm. A previous study observed that the optimal critical value of 1.35 cm to identify IAC (AUC = 0.870; sensitivity = 0.860; specificity = 0.720) [30]. In other studies, a pGGN diameter > 16.4 mm was more likely to be an IAC (sensitivity = 0.610, specificity = 0.790) [31]. These data were similar to ours. Our study showed that the IAC group had a significantly higher CT-LAP (median; − 560.94 vs. -620.45, *P* = 0.004). The AUC of CT-LAP was 0.666 and the critical value was − 597.45HU (sensitivity = 0.698, specificity = 0.638). In a previous report [32], pGGNs > 10.5 mm (AUC = 0.841; sensitivity = 0.871, specificity = 0.709), and with an attenuation > − 632 HU (AUC = 0.724; sensitivity = 0.788; specificity = 0.598) were more likely to be IAC.

We extracted radiomics features from CT images, and established a preoperative radiomics signature to identify patients with P-pGGNs at increased risk of IAC. This radiomics signature united “Sphericity”, “Small Dependence High Gray Level Emphasis”, “Joint Average”, “Imc1”, “Skewness”, “Gray Level Variance 1”, and “Long Run Emphasis”. Previous several studies have analyzed radiomics features in the diagnosis of pGGNs. Zhang et al. [33] demonstrated that histogram parameters, combined with an evaluation of morphological characteristics, exhibited good diagnostic performances in discriminating AIS/MIA from IAC, appearing as pGGNs, The AUC, sensitivity and specificity of the predictive model was 0.896, 0.794, and 0.914, respectively. Similarly, for the prediction between AIS/MIA and IAC representing as pGGNs, Xu et al. [34] showed the predictive radiomics models built in study (AUC 0.833;95% CI, 0.733–0.934) which provided a good predictive power. Besides, Sun et al. [35] developed a radiomics-based Rad-score utilized as a biomarker for the invasiveness-predicted evaluation in patients with pGGNs (AUC 0.72; 95% CI, 0.63–0.81). Their study confirmed the advantage of radiomics in the diagnosis of Benign/AAH/AIS from MIA/IAC. In another study, Song et al. [36] selected 102 radiomics features to construct the model for discrimination of AAH/AIS from MIA/IAC, which improved the good discriminative power (AUC,0.911;95%CI,0.730–0.980,Sensitivity,0.813,Specificity,0.854), significantly. Several studies have evaluated CT radiomics features for predicting invasiveness, however, due to relatively high heterogeneity and pathologically mixed grouping of enrolled lesions the results might be highly estimated when their model applied to a more detailed forecast of invasive adenocarcinoma in pGGNs. In our study, a particular group, which all have the same malignant radiological signs, was analyzed in a more detailed grouping. We determined seven features from TSCT images of P-pGGNs in our diagnostic model to identify IAC and MIA. The AUC, sensitivity and specificity were 0.892, 0.811 and 0.719, respectively. These exhibited a better discriminative performance when compared with conventional quantitative CT parameters, such as TLD alone, the AUC of which was 0.724 (sensitivity = 0.792; specificity = 0.553).

There were some limitations to our study. Firstly, this was a single center retrospective study, and therefore, additional studies are required to externally validate this model. Secondly, our study used retrospective imaging datasets, and did not take account of scanning device types, convolution kernels, reconstruction algorithms, and slice thickness. These factors could affect radiomics features, and thus, critically alter the accuracy of radiomics signatures. Additionally, our radiomics features were derived from the results of manual segmentation. Three-dimensional tumor segmentation is a complicated and time-consuming procedure. We sought to steer clear of small internal vessels and the bronchi, however, the remaining vessels may still affect the accuracy of some features. Furthermore, we did not establish a clinical model in this study. The analysis of isolated texture features and absorption of clinical parameters may facilitate further performance development of the radiomics-based prediction model. In future refinement studies, further comparative examinations of pleural contact signs in lung lesions will be performed between pathology and radiomics settings.

## Conclusions

Our radiomics method revealed lung nodules in a non-invasive manner, enabling the identification of imaging phenotypes to decode lung nodules. These P-pGGNs would receive an appropriate classification promptly, keeping away from the blind and extensive radical treatment. Our radiomics signature provided added diagnostic value to differentiate IAC from MIA in P-pGGNs and offered crucial reference can instruct follow up and prognosis prediction. What’s more, this quantitative prediction model based on the radiomic features of CT imaging, might have broader clinical applications and accelerates the development of personalized medicine, especially for the treatment of patients with lung pGGNs.

## Supplementary Information

**Additional file 1.**

## Data Availability

The datasets used and/or analyzed during the current study are available from the corresponding author on reasonable request.

## Abbreviations

pGGNs: Pure ground-glass nodules
P-pGGNs: pGGNs with pleural contact
AIS: Adenocarcinoma in situ
MIA: Minimally invasive adenocarcinoma
IAC: Invasive adenocarcinoma
IASLC, ATS, ERS: International Association for Study of Lung Cancer, American, Thoracic Society and the European Respiratory Society
WHO: World Health Organization
CT: Computed tomography
TSCT: Thin-slice computed tomography
HE: Hematoxylin/Eosin
PACS: picture archiving and communication system
HU: Hounsfield units
ROI: Region of Interest
LA: Largest axial plane
TLD: Tumor length diameter
TSD: Tumor short diameter
TVD: Tumor vertical diameter
CT-LAP: CT value on the LAP
NLD-LAP: Normal lung density measured on the same plane with LAP
RCT-LAP: Relative CT value on the LAP
LASSO: Least absolute shrinkage and selection operator
ROC: Receiver-operator characteristic
AUC: area under the ROC curve
DCA: Decision curve analyses
GLDM: Gray Level Dependence Matrix
GLCM: Gray Level Co-occurrence Matrix
GLRLM: Gray Level Run Length Matrix
GLSZM: Gray Level Size Zone Matrix
NGTDM: Neighboring Gray Tone Difference Matrix
ICC: Intra- and inter-class correlation coefficient
