# Supplementary material for: Radiomic signature based on CT imaging to distinguish invasive adenocarcinoma from minimally invasive adenocarcinoma in pure ground-glass nodules with pleural contact
Source: Cancer Imaging. 2021 Jan 6;21:1. doi: 10.1186/s40644-020-00376-1 (PMC7788838; doi:10.1186/s40644-020-00376-1)
Supplement: Supplementary file 1 — Additional file 1. [file 40644_2020_376_MOESM1_ESM.docx]

Shape features include descriptors of the three-dimensional size and shape of the ROI calculated on the non-derived image and mask. Gray Level Dependence Matrix (GLDM)features quantify the gray level dependencies in an image. Gray Level Co-occurrence Matrix (GLCM) features include descriptors of the second-order joint probability function of an image region constrained by the mask. First-order features include descriptors of the distribution of voxel intensities within the ROI adopting commonly used and basic metrics. Gray Level Run Length Matrix (GLRLM) include descriptors of quantitative gray level runs, which are defined as the length in number of pixels, of consecutive pixels that have the same gray level value. Gray Level Size Zone (GLSZM) include descriptors of gray level zones in an image. A gray level zone is defined as a number of connected voxels that share the same gray level intensity. Neighbouring Gray Tone Difference Matrix (NGTDM) quantify the difference between a gray value and the average gray value of its neighbours within distance δ.

**1、Shape Features (3D)**

In this group of features we included descriptors of the three-dimensional size and shape of the ROI. These features are independent from the gray level intensity distribution in the ROI and are therefore only calculated on the non-derived image and mask.

Unless otherwise specified, features are derived from the approximated shape defined by the triangle mesh. To build this mesh, vertices (points) are first defined as points halfway on an edge between a voxel included in the ROI and one outside the ROI. By connecting these vertices a mesh of connected triangles is obtained, with each triangle defined by 3 adjacent vertices, which shares each side with exactly one other triangle.

This mesh is generated using a marching cubes algorithm. In this algorithm, a 2x2 cube is moved through the mask space. For each position, the corners of the cube are then marked ‘segmented’ (1) or ‘not segmented’ (0). Treating the corners as specific bits in a binary number, a unique cube-index is obtained (0-255). This index is then used to determine which triangles are present in the cube, which are defined in a lookup table.

These triangles are defined in such a way, that the normal (obtained from the cross product of vectors describing 2 out of 3 edges) are always oriented in the same direction. For 3Dslicer, the calculated normals are always pointing outward. This is necessary to obtain the correct signed volume used in calculation of Mesh Volume.

Let:

$N_{v}$represent the number of voxels included in the ROI

$N_{f}$represent the number of faces (triangles) defining the Mesh.

$V$ the volume of the mesh in mm3, calculated by $get Mesh Volume Feature Value()$

$A$ the surface area of the mesh in mm2, calculated by $get Mesh Surface Area Feature Value()$

- The Sphericity was calculated as:

$$sphericity=\frac{\sqrt[3]{36\pi V^{2}}}{A}$$

Sphericity is a measure of the roundness of the shape of the tumor region relative to a sphere. It is a dimensionless measure, independent of scale and orientation. The value range is $0<sphericity\leq1$, where a value of 1 indicates a perfect sphere (a sphere has the smallest possible surface area for a given volume, compared to other solids).

**2、Gray Level Dependence Matrix (GLDM) Features**

A Gray Level Dependence Matrix (GLDM) quantifies gray level dependencies in an image. A gray level dependency is defined as a number of connected voxels within distance δ that are dependent on the center voxel. A neighbouring voxel with gray level j is considered dependent on center voxel with gray level $i$ if $|i-j|\leq\alpha$. In a gray level dependence matrix $\mathbf{P}(i,j)$ the $\left( i,j \right)^{th}$ element describes the number of times a voxel with gray level $i$ with $j$ dependent voxels in its neighbourhood appears in image.

As a two-dimensional example, consider the following 5x5 image, with 5 discrete gray levels:

$$I=\left[ \begin{matrix} \begin{matrix} \begin{matrix} 5 \\ 3 \\ \begin{matrix} 2 \\ 4 \\ 3 \end{matrix} \end{matrix} & \begin{matrix} 2 \\ 3 \\ \begin{matrix} 1 \\ 2 \\ 5 \end{matrix} \end{matrix} \end{matrix} & \begin{matrix} 5 \\ 3 \\ \begin{matrix} 1 \\ 2 \\ 3 \end{matrix} \end{matrix} & \begin{matrix} \begin{matrix} 4 \\ 1 \\ \begin{matrix} 1 \\ 2 \\ 3 \end{matrix} \end{matrix} & \begin{matrix} 4 \\ 3 \\ \begin{matrix} 3 \\ \begin{matrix} 3 \\ 2 \end{matrix} \end{matrix} \end{matrix} \end{matrix} \end{matrix} \right]$$

For$\alpha=0$and$\delta=1$, the GLDM then becomes:

$$P=\left[ \begin{matrix} \begin{matrix} 0 & 1 \end{matrix} & \begin{matrix} 2 & 1 \end{matrix} \\ \begin{matrix} \begin{matrix} 1 \\ 1 \\ \begin{matrix} 1 \\ 3 \end{matrix} \end{matrix} & \begin{matrix} 2 \\ 4 \\ \begin{matrix} 2 \\ 0 \end{matrix} \end{matrix} \end{matrix} & \begin{matrix} \begin{matrix} 3 \\ 4 \\ \begin{matrix} 0 \\ 0 \end{matrix} \end{matrix} & \begin{matrix} 0 \\ 0 \\ \begin{matrix} 0 \\ 0 \end{matrix} \end{matrix} \end{matrix} \end{matrix} \right]$$

Let:

$N_{g}$ be the number of discreet intensity values in the image

$N_{d}$ be the number of discreet dependency sizes in the image

$N_{z}$ be the number of dependency zones in the image, which is equal to $\sum{N_{g} \atop i=1}\sum{N_{d} \atop i=1}P\left( i,j \right)$

$P(i,j)$ be the dependence matrix

$p(i,j)$be the normalized dependence matrix, defined as $p(i,j)$=$\frac{P(i,j)}{N_{z}}$

- Small Dependence High Gray Level Emphasis (SDHGLE)

Measures the joint distribution of small dependence with higher gray-level values.

**3、Gray Level Co-occurrence Matrix (GLCM) Features：**

A Gray Level Co-occurrence Matrix (GLCM) of size $N_{g}\times N_{g}$ describes the second-order joint probability function of an image region constrained by the mask and is defined as $P(i,j|\delta,\theta)$. The ${(i,j)}^{\mathrm{th}}$ element of this matrix represents the number of times the combination of levels $i$and $j$ occur in two pixels in the image, that are separated by a distance of $\delta$ pixels along angle $\theta$. The distance $\delta$from the center voxel is defined as the distance according to the infinity norm. For $\delta=1$, this results in 2 neighbors for each of 13 angles in 3D (26-connectivity) and for $\delta=2$a 98-connectivity (49 unique angles).

Note that pyradiomics by default computes symmetrical GLCM!

As a two dimensional example, let the following matrix $I$ represent a 5x5 image, having 5 discrete grey levels:

$$I=\left[ \begin{matrix} \begin{matrix} \begin{matrix} 1 \\ 3 \\ \begin{matrix} 1 \\ 1 \\ 1 \end{matrix} \end{matrix} & \begin{matrix} 2 \\ 2 \\ \begin{matrix} 3 \\ 1 \\ 2 \end{matrix} \end{matrix} \end{matrix} & \begin{matrix} 5 \\ 1 \\ \begin{matrix} 5 \\ 1 \\ 4 \end{matrix} \end{matrix} & \begin{matrix} \begin{matrix} 2 \\ 3 \\ \begin{matrix} 5 \\ 1 \\ 3 \end{matrix} \end{matrix} & \begin{matrix} 3 \\ 1 \\ \begin{matrix} 2 \\ \begin{matrix} 2 \\ 5 \end{matrix} \end{matrix} \end{matrix} \end{matrix} \end{matrix} \right]$$

For distance $\delta=1$ (considering pixels with a distance of 1 pixel from each other) and angle $\theta=0^{\circ}$(horizontal plane, i.e. voxels to the left and right of the center voxel), the following symmetrical GLCM is obtained:

$$P=\left[ \begin{matrix} \begin{matrix} \begin{matrix} 6 \\ 4 \\ \begin{matrix} 3 \\ 0 \\ 0 \end{matrix} \end{matrix} & \begin{matrix} 4 \\ 0 \\ \begin{matrix} 2 \\ 1 \\ 3 \end{matrix} \end{matrix} \end{matrix} & \begin{matrix} 3 \\ 2 \\ \begin{matrix} 0 \\ 1 \\ 2 \end{matrix} \end{matrix} & \begin{matrix} \begin{matrix} 0 \\ 1 \\ \begin{matrix} 1 \\ 0 \\ 0 \end{matrix} \end{matrix} & \begin{matrix} 0 \\ 3 \\ \begin{matrix} 2 \\ \begin{matrix} 0 \\ 2 \end{matrix} \end{matrix} \end{matrix} \end{matrix} \end{matrix} \right]$$

The Sphericity was calculated as:

Let:

ϵ be an arbitrarily small positive number$(\approx2.2\times{10}^{-16})$

$P(i,j)$be the co-occurence matrix for an arbitrary $\delta$and$\theta$

$p(i,j)$ be the normalized co-occurence matrix and equal to $\frac{P(i,j)}{\sum P(i,j)}$

$N_{g}$ be the number of discrete intensity levels in the image

$p_{x}\left（ i \right）=\sum{N_{g} \atop j=1}P（i，j）$be the marginal row probabilities

$p_{y}\left（ j \right）=\sum{N_{g} \atop i=1}P（i，j）$ be the marginal column probabilities

$\mu_{x}$ be the mean gray level intensity of $p_{x}$ and defined as

$$\mu_{x}=\sum_{i=1}^{N_{g}} p_{x}（i）i$$

$\mu_{y}$ be the mean gray level intensity of py and defined as

$$\mu_{y}=\sum_{j=1}^{N_{g}} p_{y}\left( j \right)j$$

- $\sigma_{x}$ be the standard deviation of $p_{x}$
- $\sigma_{y}$ be the standard deviation of$p_{y}$
- $p_{x+y}\left（ k \right）=\sum{N_{g} \atop i=1}\sum{N_{g} \atop j=1}P\left（ i，j \right）$，where $i+j=k$, and $k=2,3,\ldots,2N_{g}$
- $p_{x-y}\left（ k \right）=\sum{N_{g} \atop i=1}\sum{N_{g} \atop j=1}P\left（ i，j \right）$，where $\left| i-j \right|=k$, and $k=0,1,\ldots,N_{g}-1$
- $HX=- \sum{N_{g} \atop i=1}p_{x}\left（ i \right）\log_{2} \left（ p_{x}\left（ i \right）+\epsilon\right）$be the entropy of $p_{x}$
- $HY=- \sum{N_{g} \atop j=1}p_{y}\left（ j \right）\log_{2} \left（ p_{y}\left（ j \right）+\epsilon\right）$be the entropy of $p_{y}$
- $HXY=- \sum{N_{g} \atop i=1}\sum{N_{g} \atop j=1}p\left（ i,j \right）\log_{2} \left（ p\left（ i,j \right）+\epsilon\right）$be the entropy of$p\left( i,j \right)$
- $HXY1=- \sum{N_{g} \atop i=1}\sum{N_{g} \atop j=1}p\left（ i,j \right）\log_{2} \left（ p_{x}\left（ i \right）p_{y}\left( j \right)+\epsilon\right）$
- $HXY2=- \sum{N_{g} \atop i=1}\sum{N_{g} \atop j=1}p_{x}\left（ i \right）p_{y}\left（ j \right）\log_{2} \left（ p_{x}\left（ i \right）p_{y}\left( j \right)+\epsilon\right）$

By default, the value of a feature is calculated on the GLCM for each angle separately, after which the mean of these values is returned. If distance weighting is enabled, GLCM matrices are weighted by weighting factor W and then summed and normalised. Features are then calculated on the resultant matrix. Weighting factor W is calculated for the distance between neighbouring voxels by:

$W=e^{{-\left\| d \right\|}^{2}}$, where d is the distance for the associated angle according to the norm specified in setting ‘weightingNorm’.

The following class specific settings are possible:

- distances [[1]]: List of integers. This specifies the distances between the center voxel and the neighbor, for which angles should be generated.
- Symmetrical GLCM [True]: boolean, indicates whether co-occurrences should be assessed in two directions per angle, which results in a symmetrical matrix, with equal distributions for $i$ and$j$. A symmetrical matrix corresponds to the GLCM as defined by Haralick et al.
- weightingNorm [None]: string, indicates which norm should be used when applying distance weighting. Enumerated setting, possible values:
- ‘manhattan’: first order norm
- ‘euclidean’: second order norm
- ‘infinity’: infinity norm.
- ‘no_weighting’: GLCMs are weighted by factor 1 and summed
- None: Applies no weighting, mean of values calculated on separate matrices is returned.

In case of other values, an warning is logged and option ‘no_weighting’ is used.

- The Joint Average was calculated as:

$$joint average=\sum_{i=1}^{N_{g}} \sum_{j=1}^{n} p(i,j)i$$

Returns the mean gray level intensity of the$i$ distribution.

- The IMC1 was calculated as：

$$IMC1=\frac{HXY-HXY1}{max\{HX,HY\}}$$

IMC1 assesses the correlation between the probability distributions of $i$ and$j$ (quantifying the complexity of the texture), using mutual information I(x, y):

$$I\left( i,j \right)=\sum_{i=1}^{N_{g}} \sum_{j=1}^{N_{g}} p(i,j)\log_{2} \left( \frac{p(i,j)}{p_{x}(i)p_{y}(j)} \right)$$

$$=\sum_{i=1}^{N_{g}} \sum_{j=1}^{N_{g}} p(i,j)(\log_{2} \left( p\left( i,j \right) \right)-\log_{2} \left( p_{x}(i)p_{y}\left( j \right)) \right)$$

$$=\sum_{i=1}^{N_{g}} \sum_{j=1}^{N_{g}} p(i,j)\log_{2} \left( p\left( i,j \right) \right)-\sum_{i=1}^{N_{g}} \sum_{j=1}^{N_{g}} p(i,j)(\log_{2} \left( p_{x}\left( i \right)p_{y}(j) \right)$$

$$=-HXY+HXY1$$

However, in this formula, the numerator is defined as $HXY - HXY1 (i.e. -I(x,y))$, and is therefore ≤0. This reflects how this feature is defined in the original Haralick paper.

In the case where the distributions are independent, there is no mutual information and the result will therefore be 0. In the case of uniform distribution with complete dependence, mutual information will be equal to $\log_{2} N_{g}$.

Finally,$HXY-HXY1$ is divided by the maximum of the 2 marginal entropies, where in the latter case of complete dependence (not necessarily uniform; low complexity) it will result in $IMC1=-1$, as $HX=HY=I(i,j)$.

4、**First Order Features**

First-order statistics describe the distribution of voxel intensities within the image region defined by the mask through commonly used and basic metrics.

Let:

$X$ be a set of $N_{p}$ voxels included in the ROI

$P(i)$ be the first order histogram with $N_{g}$ discrete intensity levels, where $N_{g}$ is the number of non-zero bins, equally spaced from 0 with a width defined in the （binWidth） parameter.

p(i) be the normalized first order histogram and equal to $\frac{P(i)}{N_{p}}$

Following additional settings are possible:

voxelArrayShift [0]: Integer, This amount is added to the gray level intensity in features Energy, Total Energy and RMS, this is to prevent negative values. If using CT data, or data normalized with mean 0, consider setting this parameter to a fixed value (e.g. 2000) that ensures non-negative numbers in the image. Bear in mind however, that the larger the value, the larger the volume confounding effect will be.

- The Skewness was calculated as:

$$skewness=\frac{\mu_{3}}{\sigma^{3}}=\frac{\frac{1}{N_{P}}\sum_{i=1}^{N_{p}} {(X(i)-\bar{X})}^{3}}{\left( \sqrt{\frac{1}{N_{P}}\sum_{i=1}^{N_{p}} \left( X\left( i \right)-\bar{X} \right)^{2}} \right)^{3}}$$

Where $\mu_{3}$is the $3^{rd}$central moment.

Skewness measures the asymmetry of the distribution of values about the Mean value. Depending on where the tail is elongated and the mass of the distribution is concentrated, this value can be positive or negative.

5、**Gray Level Run Length Matrix (GLRLM) Features**

A Gray Level Run Length Matrix (GLRLM) quantifies gray level runs, which are defined as the length in number of pixels, of consecutive pixels that have the same gray level value. In a gray level run length matrix$P(i,j|\theta)$, the${(i,j)}^{th}$ element describes the number of runs with gray level$i$ and length $j$ occur in the image (ROI) along angle $\theta$.

As a two dimensional example, consider the following 5x5 image, with 5 discrete gray levels:

$$I=\left[ \begin{matrix} \begin{matrix} \begin{matrix} 5 \\ 3 \\ \begin{matrix} 2 \\ 4 \\ 3 \end{matrix} \end{matrix} & \begin{matrix} 2 \\ 3 \\ \begin{matrix} 1 \\ 2 \\ 5 \end{matrix} \end{matrix} \end{matrix} & \begin{matrix} 5 \\ 3 \\ \begin{matrix} 1 \\ 2 \\ 3 \end{matrix} \end{matrix} & \begin{matrix} \begin{matrix} 4 \\ 1 \\ \begin{matrix} 1 \\ 2 \\ 3 \end{matrix} \end{matrix} & \begin{matrix} 4 \\ 3 \\ \begin{matrix} 3 \\ \begin{matrix} 3 \\ 2 \end{matrix} \end{matrix} \end{matrix} \end{matrix} \end{matrix} \right]$$

The GLRLM for θ=0, where 0 degrees is the horizontal direction, then becomes:

$$P=\left[ \begin{matrix} \begin{matrix} \begin{matrix} 1 \\ 3 \\ \begin{matrix} 4 \\ 1 \\ 3 \end{matrix} \end{matrix} & \begin{matrix} 0 \\ 0 \\ \begin{matrix} 1 \\ 1 \\ 0 \end{matrix} \end{matrix} \end{matrix} & \begin{matrix} 1 \\ 1 \\ \begin{matrix} 1 \\ 0 \\ 0 \end{matrix} \end{matrix} & \begin{matrix} \begin{matrix} 0 \\ 0 \\ \begin{matrix} 0 \\ 0 \\ 0 \end{matrix} \end{matrix} & \begin{matrix} 0 \\ 0 \\ \begin{matrix} 0 \\ \begin{matrix} 0 \\ 0 \end{matrix} \end{matrix} \end{matrix} \end{matrix} \end{matrix} \right]$$

Let:

- $N_{g}$ be the number of discreet intensity values in the image
- $N_{r}$be the number of discreet run lengths in the image
- $N_{p}$ be the number of voxels in the imag
- $N_{r}\left( \theta\right)$ be the number of runs in the image along angle $\theta$, which is equal to $\sum{N_{g} \atop i=1}\sum{N_{r} \atop j=1}P\left（ \left. i，j \right|\theta\right）$and $1\leq N_{r}\left( \theta\right)\leq N_{p}$
- $P\left( i,j | \theta\right)$ be the run length matrix for an arbitrary direction $\theta$
- $p(i,j|\theta)$ be the normalized run length matrix, defined as$p(i,j|\theta)=\frac{P(i,j|\theta)}{N_{r}(\theta)}$

By default, the value of a feature is calculated on the GLRLM for each angle separately, after which the mean of these values is returned. If distance weighting is enabled, GLRLMs are weighted by the distance between neighbouring voxels and then summed and normalised. Features are then calculated on the resultant matrix. The distance between neighbouring voxels is calculated for each angle using the norm specified in ‘weightingNorm’.

The following class specific settings are possible:

- weightingNorm [None]: string, indicates which norm should be used when applying distance weighting. Enumerated setting, possible values:
- ‘manhattan’: first order norm
- ‘euclidean’: second order norm
- ‘infinity’: infinity norm.
- ‘no_weighting’: GLCMs are weighted by factor 1 and summed
- None: Applies no weighting, mean of values calculated on separate matrices is returned.

In case of other values, an warning is logged and option ‘no_weighting’ is used.

- The Gray Level Variance (GLV) was calculated as:

$$GLV=\sum_{i=1}^{N_{g}} \sum_{j=1}^{N_{g}} p(i,j){(i-\mu)}^{2}$$

Here,

$$\mu=\sum_{i=1}^{N_{g}} \sum_{j=1}^{N_{S}} p(i,j)i$$

GLV measures the variance in gray level intensity for the runs.

- The Long Run Emphasis (LRE) was calculated as:

$$LRE=\frac{\sum_{i=1}^{N_{g}} \sum_{j=1}^{N_{r}} P(i,j|\theta)j^{2}}{N_{r}(\theta)}$$

LRE is a measure of the distribution of long run lengths, with a greater value indicative of longer run lengths and more coarse structural textures.

**Table 1.** Inter- and intra-class correlation coefficient of radiomics feature

|  | Radiomics feature | Inter-class correlation coefficient (95% CI) | Intra-class correlation coefficient (95% CI) |
| --- | --- | --- | --- |
| shape | Maximum3DDiameter | 0.954(0.922-0.973) | 0.934(0.889-0.961) |
|  | Maximum2DDiameterSlice | 0.942(0.902-0.966) | 0.933(0.888-0.961) |
|  | Sphericity | 0.820(0.707-0.892) | 0.776(0.643-0.864) |
|  | MinorAxis | 0.958(0.929-0.976) | 0.957(0.927-0.975) |
|  | Elongation | 0.830(0.723-0.899) | 0.881(0.803-0.929) |
|  | SurfaceVolumeRatio | 0.782(0.650-0.868) | 0.913(0.854-0.949) |
|  | Volume | 0.966(0.942-0.980) | 0.968(0.946-0.982) |
|  | MajorAxis | 0.974(0.956-0.985) | 0.963(0.937-0.978) |
|  | SurfaceArea | 0.964(0.939-0.979) | 0.943(0.904-0.967) |
|  | Flatness | 0.792(0.665-0.874) | 0.887(0.812-0.933) |
|  | LeastAxis | 0.969(0.947-0.982) | 0.942(0.903-0.966) |
|  | Maximum2DDiameterColumn | 0.959(0.929-0.976) | 0.948(0.912-0.970) |
|  | Maximum2DDiameterRow | 0.953(0.920-0.973) | 0.929(0.880-0.958) |
| gldm | GrayLevelVariance | 0.801(0.678-0.880) | 0.905(0.842-0.944) |
|  | HighGrayLevelEmphasis | 0.951(0.916-0.971) | 0.949(0.913-0.970) |
|  | DependenceEntropy | 0.711(0.547-0.822) | 0.821(0.710-0.892) |
|  | DependenceNonUniformity | 0.926(0.875-0.957) | 0.951(0.916-0.971) |
|  | GrayLevelNonUniformity | 0.983(0.971-0.990) | 0.981(0.967-0.989) |
|  | SmallDependenceEmphasis | 0.886(0.810-0.932) | 0.982(0.970-0.990) |
|  | SmallDependenceHighGrayLevelEmphasis | 0.949(0.914-0.971) | 0.959(0.930-0.976) |
|  | DependenceNonUniformityNormalized | 0.883(0.806-0.931) | 0.986(0.976-0.992) |
|  | LargeDependenceEmphasis | 0.926(0.875-0.956) | 0.989(0.981-0.993) |
|  | LargeDependenceLowGrayLevelEmphasis | 0.287(0.021-0.516) | 0.744(0.596-0.844) |
|  | DependenceVariance | 0.918(0.863-0.952) | 0.981(0.968-0.989) |
|  | LargeDependenceHighGrayLevelEmphasis | 0.896(0.827-0.939) | 0.917(0.861-0.951) |
|  | SmallDependenceLowGrayLevelEmphasis | 0.506(0.276-0.682) | 0.601(0.399-0.748) |
|  | LowGrayLevelEmphasis | 0.514(0.285-0.687) | 0.633(0.441-0.770) |
| glcm | JointAverage | 0.938(0.895-0.964) | 0.941(0.901-0.966) |
|  | SumAverage | 0.938(0.895-0.964) | 0.941(0.901-0.966) |
|  | JointEntropy | 0.866(0.778-0.875) | 0.881(0.802-0.929) |
|  | ClusterShade | 0.463(0.222-0.650) | 0.746(0.598-0.845) |
|  | MaximumProbability | 0.828(0.719-0.897) | 0.890(0.818-0.935) |
|  | Idmn | 0.630(0.436-0.768) | 0.822(0.711-0.893) |
|  | JointEnergy | 0.842(0.742-0.906) | 0.914(0.857-0.95) |
|  | Contrast | 0.767(0.628-0.858) | 0.946(0.909-0.969) |
|  | DifferenceEntropy | 0.855(0.761-0.914) | 0.947(0.911-0.969) |
|  | InverseVariance | 0.879(0.799-0.928) | 0.973(0.954-0.984) |
|  | DifferenceVariance | 0.753(0.607-0.849) | 0.926(0.876-0.957) |
|  | Idn | 0.671(0.492-0.796) | 0.892(0.723-0.898) |
|  | Idm | 0.893(0.822-0.937) | 0.974(0.955-0.985) |
|  | Correlation | 0.659(0.475-0.788) | 0.815(0.700-0.888) |
|  | Autocorrelation | 0.943(0.904-0.967) | 0.949(0.914-0.970) |
|  | SumEntropy | 0.833(0.727-0.900) | 0.874(0.792-0.925) |
|  | MCC | 0.674(0.495-0.798) | 0.687(0.514-0.806) |
|  | SumSquares | 0.764(0.623-0.856) | 0.896(0.827-0.939) |
|  | ClusterProminence | 0.446(0.202-0.638) | 0.811(0.695-0.886) |
|  | Imc2 | 0.894(0.823-0.937) | 0.953(0.921-0.973) |
|  | Imc1 | 0.810(0.692-0.886) | 0.949(0.914-0.970) |
|  | DifferenceAverage | 0.823(0.712-0.894) | 0.961(0.934-0.977) |
|  | Id | 0.883(0.806-0.931) | 0.973(0.953-0.984) |
|  | ClusterTendency | 0.751(0.604-0.848) | 0.872(0.789-0.924) |
| firstorder | InterquartileRange | 0.862(0.772-0.918) | 0.910(0.849-0.947) |
|  | Skewness | 0.782(0.650-0.868) | 0.853(0.759-0.912) |
|  | Uniformity | 0.887(0.811-0.933) | 0.941(0.900-0.965) |
|  | Median | 0.946(0.909-0.969) | 0.979(0.963-0.988) |
|  | Energy | 0.952(0.919-0.971) | 0.962(0.936-0.978) |
|  | RobustMeanAbsoluteDeviation | 0.861(0.771-0.918) | 0.914(0.856-0.949) |
|  | MeanAbsoluteDeviation | 0.856(0.763-0.914) | 0.920(0.865-0.953) |
|  | TotalEnergy | 0.967(0.943-0.981) | 0.972(0.952-0.984) |
|  | Maximum | 0.726(0.569-0.832) | 0.656(0.472-0.786) |
|  | RootMeanSquared | 0.943(0.904-0.967) | 0.971(0.951-0.983) |
|  | 90Percentile | 0.941(0.900-0.966) | 0.982(0.968-0.989) |
|  | Minimum | 0.824(0.714-0.895) | 0.720(0.561-0.828) |
|  | Entropy | 0.889(0.816-0.935) | 0.932(0.886-0.960) |
|  | Range | 0.752(0.606-0.849) | 0.629(0.435-0.767) |
|  | Variance | 0.800(0.678-0.880) | 0.905(0.842-0.944) |
|  | 10Percentile | 0.831(0.724-0.899) | 0.851(0.755-0.911) |
|  | Kurtosis | 0.774(0.639-0.863) | 0.813(0.698-0.888) |
|  | Mean | 0.950(0.914-0.971) | 0.978(0.963-0.987) |
| glrlm | ShortRunLowGrayLevelEmphasis | 0.528(0.303-0.698) | 0.623(0.428-0.763) |
|  | GrayLevelVariance | 0.800(0.677-0.879) | 0.904(0.839-0.943) |
|  | LowGrayLevelRunEmphasis | 0.524(0.298-0.695) | 0.625(0.430-0.764) |
|  | GrayLevelNonUniformityNormalized | 0.886(0.810-0.993) | 0.940(0.899-0.965) |
|  | RunVariance | 0.913(0.854-0.949) | 0.988(0.979-0.993) |
|  | GrayLevelNonUniformity | 0.982(0.969-0.990) | 0.979(0.964-0.988) |
|  | LongRunEmphasis | 0.917(0.860-0.951) | 0.989(0.980-0.993) |
|  | ShortRunHighGrayLevelEmphasis | 0.951(0.917-0.972) | 0.949(0.913-0.970) |
|  | RunLengthNonUniformity | 0.943(0.903-0.967) | 0.956(0.925-0.974) |
|  | ShortRunEmphasis | 0.917(0.861-0.951) | 0.989(0.980-0.993) |
|  | LongRunHighGrayLevelEmphasis | 0.946(0.909-0.969) | 0.942(0.901-0.966) |
|  | RunPercentage | 0.915(0.857-0.950) | 0.989(0.981-0.994) |
|  | LongRunLowGrayLevelEmphasis | 0.501(0.269-0.678) | 0.629(0.436-0.767) |
|  | RunEntropy | 0.853(0.758-0.912) | 0.889(0.816-0.934) |
|  | HighGrayLevelRunEmphasis | 0.950(0.916-0.971) | 0.947(0.911-0.969) |
|  | RunLengthNonUniformityNormalized | 0.915(0.857-0.950) | 0.988(0.980-0.993) |
| glszm | GrayLevelVariance | 0.750(0.603-0.847) | 0.852(0.758-0.910) |
|  | ZoneVariance | 0.991(0.984-0.995) | 0.995(0.992-0.997) |
|  | GrayLevelNonUniformityNormalized | 0.864(0.775-0.919) | 0.915(0.857-0.950) |
|  | SizeZoneNonUniformityNormalized | 0.832(0.726-0.900) | 0.923(0.870-0.955) |
|  | SizeZoneNonUniformity | 0.920(0.865-0.953) | 0.950(0.916-0.971) |
|  | GrayLevelNonUniformity | 0.960(0.931-0.976) | 0.950(0.915-0.971) |
|  | LargeAreaEmphasis | 0.991(0.984-0.995) | 0.995(0.992-0.997) |
|  | SmallAreaHighGrayLevelEmphasis | 0.935(0.889-0.962) | 0.917(0.862-0.951) |
|  | ZonePercentage | 0.888(0.814-0.934) | 0.985(0.975-0.991) |
|  | LargeAreaLowGrayLevelEmphasis | 0.992(0.986-0.995) | 0.990(0.984-0.994) |
|  | LargeAreaHighGrayLevelEmphasis | 0.987(0.978-0.993) | 0.995(0.992-0.997) |
|  | HighGrayLevelZoneEmphasis | 0.934(0.889-0.962) | 0.920(0.866-0.953) |
|  | SmallAreaEmphasis | 0.832(0.726-0.899) | 0.918(0.862-0.952) |
|  | LowGrayLevelZoneEmphasis | 0.567(0.352-0.725) | 0.501(0.271-0.678) |
|  | ZoneEntropy | 0.683(0.508-0.804) | 0.817(0.704-0.890) |
|  | SmallAreaLowGrayLevelEmphasis | 0.531(0.306-0.699) | 0.476(0.239-0.659) |
| ngtdm | Coarseness | 0.787(0.657-0.871) | 0.920(0.866-0.953) |
|  | Complexity | 0.772(0.636-0.862) | 0.832(0.727-0.900) |
|  | Strength | 0.830(0.722-0.898) | 0.900(0.834-0.941) |
|  | Contrast | 0.759(0.616-0.853) | 0.938(0.895-0.964) |
|  | Busyness | 0.955(0.923-0.974) | 0.947(0.910-0.969) |
